# Prospective associations between sedentary time, sleep duration and adiposity in adolescents

**DOI:** 10.1016/j.sleep.2015.02.532

**Published:** 2015-06

**Authors:** Paul J. Collings, Katrien Wijndaele, Kirsten Corder, Kate Westgate, Charlotte L. Ridgway, Stephen J. Sharp, Andrew J. Atkin, Diane Bamber, Ian Goodyer, Soren Brage, Ulf Ekelund

**Affiliations:** a MRC Epidemiology Unit, University of Cambridge School of Clinical Medicine, Cambridge, UK; b UKCRC Centre for Diet and Activity Research (CEDAR), MRC Epidemiology Unit, University of Cambridge School of Clinical Medicine, Cambridge, UK; c Developmental Lifecourse Research Group, Department of Psychiatry, University of Cambridge, Cambridge, UK; d Department of Sport Medicine, Norwegian School of Sports Science, Oslo, Norway

**Keywords:** Sedentariness, Sleep length, Body fatness, Obesity, Youth, Cohort study, FM, fat mass, FFM, fat-free mass, FFMI, fat-free mass index, FMI, fat mass index, METs, metabolic equivalents of thermogenesis, MVPA, moderate-to-vigorous physical activity, PAEE, physical activity energy expenditure, SES, socio-economic status, ΔFFMI, change in fat-free mass index, ΔFMI, change in fat mass index

## Abstract

**Objective:**

The objective of this study was to investigate whether objectively measured sedentary time and sleep duration are associated with changes in adiposity from mid- to late adolescence.

**Methods:**

Students (*n* = 504, 42% boys) were recruited from schools in Cambridgeshire, UK. At baseline (mean age 15.0 ± 0.3 years), sedentary time was objectively measured by ≥3 days of combined heart rate and movement sensing. Concurrently, sleep duration was measured by combined sensing in conjunction with self-reported bed times. Fat mass index (FMI; kg/m^2^) was estimated at baseline and follow-up (17.5 ± 0.3 years) by anthropometry and bioelectrical impedance. FMI change (ΔFMI) was calculated by subtracting the baseline from follow-up values. Linear regression models adjusted for basic demographics, moderate-to-vigorous physical activity (MVPA), and depressive symptoms were used to investigate associations of sedentary time and sleep duration (mutually adjusted for one another) with ΔFMI.

**Results:**

FMI increased by 0.5 and 0.6 kg/m^2^ in boys and girls, respectively, but there was no association between sedentary time and ΔFMI in either gender (*p* ≥ 0.087), and no association between sleep duration and ΔFMI in girls (*p* ≥ 0.61). In boys, each additional hour of baseline sleep significantly reduced the ΔFMI by 0.13 kg/m^2^ (*p* = 0.049), but there was little evidence for this association after adjusting for MVPA and depressive symptoms (*p* = 0.15).

**Conclusions:**

Sedentary time may not determine changes in adiposity from mid- to late adolescence, nor may sleep duration in girls. However, sleep length may be inversely associated with adiposity gain in boys, depending on whether the relationship is confounded or mediated by MVPA and depression.

## Highlights

• We examined sedentary time and sleep length relative to changes in youth adiposity.
• Sedentary time was not associated with change in adiposity in either gender.
• Sleep duration was significantly inversely associated with adiposity gain in boys.
• The association for sleep in boys was attenuated by physical activity and depression.

## 1 Introduction

Prolonged sitting is highly prevalent in modern society [1], and it is composed of numerous diverse behaviours many of which (eg, seated reading, writing, and screen viewing) but not all (seated cycling or rowing) contribute to the total time spent sedentary. There is some evidence that a secular trend of increasing sedentary time has coincided with continuing emergence of the obesity epidemic [2,3], leading to the conjecture that a causal relationship may exist. However, most aetiological investigations of sedentary time and obesity have measured TV-viewing duration only, which is an imperfect and unrepresentative proxy of the total time spent sedentary [4,5]. More studies are needed to investigate the total sedentary time, also measured more precisely by objective instead of self-report methods, and its association with obesity in youth, particularly as doubts have arisen as to whether an association exists for total sedentary time that is independent of physical activity [6].

Like sedentary time, sleep is a state of rest that involves immobile posture and low energy expenditure. However, it is further characterised by reversible complete or partial loss of consciousness and responsiveness to external stimuli. Adequate sleep is essential for normal growth, development and functioning in youth [7–9]. It is therefore concerning that sleep durations have declined over the 20th century by >1 h/night [10]. Adolescents are currently failing to reach sleep targets [9], and the prevalence estimates for adolescent fatigue have increased since the mid-1980s [11]. Speculatively, short sleep durations may be implicated in the aetiology of obesity. Supporting this theory, laboratory studies in adults have shown that restricted sleep deregulates endocrine secretions, causing decreased leptin and increased ghrelin, which may predispose to obesity through increased energy intake [12]. Physical activity, sedentary time, and depression have also been proposed as mediating factors [13]. However, the strength of evidence directly linking sleep length to obesity in adolescence is currently limited. The literature is dominated by cross-sectional studies, measuring sleep duration with non-validated questions and utilising body mass index (BMI) as an outcome [14,15].

The purpose of this prospective study was to incorporate objective measurements of total sedentary time and sleep duration, to investigate whether they are independently and oppositely associated with changes in body fatness from mid- to late adolescence.

## 2 Subjects and methods

### 2.1 Study design

Participants were from schools registered in the ROOTS study, an observational cohort study based in Cambridge, UK [16]. Schools (*n* = 18) were recruited from a geographical perimeter surrounding the city and extending to surrounding villages. Students within schools were eligible to take part if they were aged 14 years. At the start of the study (wave 0), 1238 students gave written informed consent to participate, with 1203 students (45% boys) eventually attending for data collection. Details about basic demographics (date of birth, gender, ethnicity and postcode to indicate socio-economic status, SES) and pubertal development were self-reported, and anthropometrics and body composition were also measured. Six months later (wave 1, from here on referred to as baseline), 930 students (43% boys) were seen for a second measurement of anthropometry and body composition, and at this time habitual activity monitoring was performed. Approximately 2.5 years later (wave 2, here referred to as follow-up), 844 students (44% boys) attended for the third and final measurements of anthropometry and body composition. All stages of the ROOTS project were approved by the Cambridge Local Research Ethics Committee (reference number 03/302) and were conducted in agreement with the Declaration of Helsinki guidance.

### 2.2 Anthropometry and body composition

Detailed physical measurements were made by trained personnel at all waves using identical procedures and instrumentation. Height was measured to the nearest 0.1 cm (Leicester Height Metre; Invicta Plastics, Leicester, UK) whilst barefoot, and weight was measured to the nearest 0.1 kg in light clothing (Tanita TBF-300 MA, Tanita, Tokyo, Japan) using standard procedures. BMI (kg/m^2^) was calculated and body tissue impedance (Ω) was measured by bio-impedance (Tanita TBF-300 MA). Subsequently, child-specific equations [17] were used to derive multiple estimates of fat mass (FM, kg) and fat-free mass (FFM, kg) by utilising the data on height, weight, BMI, and body tissue impedance (values from equations predicting total body water were converted to FFM and FM using age- and sex-specific data on the hydration of lean tissue [18]). All permutations were pooled alongside body composition measured by the Tanita TBF-300 MA to produce aggregated measures of FM and FFM [19], which were expressed relative to height-squared (fat mass index (FMI), kg/m^2^) and height raised to the power of 2.5 (fat-free mass index (FFMI), kg/m^2.5^), respectively [20].

### 2.3 Sedentary time, sleep duration, and physical activity

Habitual activity was measured objectively by combined heart rate and movement sensing (Actiheart, CamNtech Ltd, Papworth, UK) at wave 1. A detailed description of the monitoring protocol can be found elsewhere [17]. In brief, following a graded sub-maximal step test to establish individual calibration of heart rate, the sensor was initialised to record data every 30 s and was worn by participants without interruption for up to four consecutive days, including a weekend. Data from participants who had worn the sensor for at least 32 h on weekdays and 16 h on weekend days were considered useable, with a further proviso that these hours were distributed across the 24-h period (thereby providing the equivalent of ≥3 days and nights of activity monitoring).

Information collected during the free-living period were pre-processed [21] and an activity intensity (J/min/kg) estimate made by a branched equation framework [22]. Upon summarising the data, potential bias caused by non-wear periods (segments of non-physiological data) was minimised [23] and the resulting time-series data were collapsed into physical activity energy expenditure (PAEE, kJ/kg/day) and time spent in different intensity categories. Standard metabolic equivalents of thermogenesis (METs) were used to establish the time spent sedentary (≤1.5 METs) and in moderate-to-vigorous physical activity (MVPA, > 4.0 METs). To assist separation of sleep and sedentary time, participants were asked to report the times that they usually went to bed and got up on school days and weekend days separately, as defined in the Sleep Habits Survey for Adolescents, which has been validated against sleep diary and actigraphy [24]. This self-reported information was overlaid on the habitual activity data to provide a region of interest within which to identify objective markers of sleep onset (considered the beginning of prolonged minimal movement accompanied by a decline in heart rate) and termination (movement initiation together with an abrupt increase in heart rate); the self-reported sleep data were subsequently adjusted commensurate with these objective sleep indicators by reviewer visual inspection [17]. A single researcher reviewed all activity plots whilst blinded to all other participant characteristics, and every occurrence of time spent ≤1.5 METs was allocated either a sleep (=1) or awake (=0, ie, sedentary time) score depending on whether the behaviour fell within or outside of a designated sleep phase.

### 2.4 Other covariates

Home postcodes were used to create an area-level SES indicator that was collapsed from five original groups [25] to three categories: low (hard-pressed and moderate means), middle (comfortably off), and high (urban prosperity and wealthy achievers).

Puberty status (dichotomised as pre- or post-pubertal) was determined by features including self-reported menarcheal status, self-reported Tanner stages and objective levels of salivary testosterone. Girls who were post menarche at wave 0 were considered pubertal, as were pre-menarcheal girls reporting advanced signs of puberty (pubic hair or breast development ≥3 of the Tanner scale) [26]. All remaining girls were defined as pre-pubertal. Boys who reported pubic hair coverage and genital development ≥4 of the Tanner scale were classified as post-pubertal, whereas boys reporting stages ≤2 on both axes were regarded as pre-pubertal. All remaining boys were categorised according to salivary testosterone levels, with those whose level was ≥25th percentile of the distribution from the pubertal group being labelled post-pubertal.

Students at wave 0 completed the Mood and Feelings Questionnaire (MFQ), a 33-item self-report scale eliciting information about depressive symptoms occurring in the previous fortnight. The MFQ has been validated as a screening tool for clinical unipolar depression in adolescents [27]; higher summed MFQ scores are indicative of increased risk [28]. Students rated their symptoms on a four-point scale (never/sometimes/mostly/always) from which a summed score was computed and used in analyses. In the ROOTS study, internal consistency of the separate items of the MFQ has been demonstrated to be high (Cronbach's *α* = 0.96).

At 18 months post wave (a stage in the ROOTS study that was primarily used to collect adolescent psychosocial measures and is not otherwise used in this investigation), height and weight were measured in a subsample of participants' mothers. These data were used to compute maternal BMI, which was used only in sensitivity analyses due to missing data.

### 2.5 Statistics

All statistical analyses were conducted in Stata/SE 13.1 (StataCorp, College Station, TX, USA). Participant characteristics at baseline were summarised as frequencies and percentages, means with standard deviations or medians with interquartile ranges. Differences in these characteristics between boys and girls were tested using chi-squared (*χ*^2^) tests, independent *t*-tests and Wilcoxon rank sum tests. Pearson correlation was used to explore the nature of the relationships between sedentary time, sleep duration, and MVPA. Mixed-model analysis of variance (ANOVA) was used to test for differences between baseline and follow-up anthropometry and body composition; the interaction between gender and wave of data collection was used to investigate whether these differences were gender specific.

This investigation constituted a complete-case analysis involving only participants with valid exposure (sedentary time and sleep duration) information at baseline, and complete information for covariates and adiposity data at both baseline and follow-up. The following steps were taken to determine the representativeness of contributing adolescents relative to the wider ROOTS cohort: (1) participants with valid exposure data at baseline (wave 1) were compared to all adolescents who failed to provide these data with respect to their wave 0 adiposity levels, using the Wilcoxon rank sum test, and (2) a comparison of sedentary time and sleep duration levels at baseline was made using independent sample *t*-tests between contributing participants and participants who also had valid exposure data at baseline, but who were excluded from analyses due to missing covariate data or loss to follow-up (i.e., missing wave 2 adiposity).

Linear regression models were used to investigate whether baseline sedentary time or baseline sleep duration was associated with changes in FMI over time (ΔFMI = follow-up FMI – baseline FMI). Robust standard errors were calculated to account for school-level clustering. For both exposure/outcome combinations, two models were fit. Model 1 included baseline age, area-level SES, pubertal status, season of activity assessment, weekday and weekend monitor wear time and follow-up duration. Ethnicity was not included in the model due to low variation (94% of all ROOTS participants were White). Model 2 included all covariates from Model 1 as well as time spent in MVPA and depressive symptoms. Sedentary time and sleep duration were also mutually adjusted for one another in Model 2; the coefficients of this second model can be interpreted as the effect of exchanging 1 h of light physical activity for sedentary time or sleep. Adjustment for PAEE instead of MVPA was performed as part of a sensitivity analysis, as was adjustment for maternal BMI, baseline FMI, wave 0 (pre-baseline) FMI or ΔFMI from wave 0 to 1. Some studies of sleep duration and obesity (particularly in adults) have reported “U”-shaped associations [29]. Non-linear associations between sleep duration and ΔFMI were therefore investigated by introducing quadratic terms for sleep duration. The results are expressed as the change in ΔFMI per hour of baseline sedentary or sleep time. Similar models were run for FFMI.

## 3 Results

As shown in Table 1, which describes the 504 included participants with complete data (42.3% boys), the sample was homogeneous with respect to ethnicity and pubertal status, and approximately 85% of participants were from middle- to high-SES locations. Boys self-reported fewer symptoms of depression and were more physically active and less sedentary than girls, but both genders engaged in considerable sedentary time (~6 h/day). Both genders slept for around 8 h/night with boys sleeping marginally less. Sleep duration was weakly negatively correlated with MVPA (boys: *r* = −0.16; girls: *r* = −0.14) and seemingly uncorrelated with sedentary time (boys: *r* = 0.01; girls: *r* = 0.02), whereas sedentary time and MVPA were moderately negatively correlated (boys: *r* = −0.49; girls: *r* = −0.50). Compared to participants who were measured at wave 0 but who chose not to contribute to baseline (*n* = 273), or participated at baseline but failed to provide valid exposure data (*n* = 194), participants with valid sedentary time and sleep data (*n* = 736) had lower FMI at wave 0 [median (IQR): 4.2 (3.0) vs. 4.5 (3.3) kg/m^2^, *p* = 0.034]. There was no difference in the levels of sedentary time or sleep length (*p* ≥ 0.82 for both) between the 504 finally included participants and those adolescents with valid exposure data but missing covariates (*n* = 46, most missing data were for puberty and depressive symptoms) or missing follow-up adiposity (*n* = 186).

The data for anthropometry and body composition at baseline and follow-up are summarised in Table 2. Body size (weight, height, BMI, FFM and FFMI) increased in both genders over time but to a greater extent in boys than girls, maybe reflecting a higher proportion of boys transitioning from pre- to post-puberty during follow-up (at wave 0, 20% of boys were pre-pubertal vs. only 2% of girls). Fat mass and FMI also increased over follow-up, but the magnitude of change was not different between genders; boys acquired 0.5 and girls 0.6 kg/m^2^ of FMI.

Associations of baseline sedentary time and sleep duration with changes in FMI and FFMI are shown in Table 3. For FMI, there was some evidence for interactions between gender and sedentary time (*p*-gender interaction = 0.055) and gender with sleep (*p*-gender interaction = 0.078); therefore, all analyses were stratified by gender. There were no significant associations between baseline sedentary time and ΔFMI in either boys or girls, and there was no significant association between baseline sleep duration and ΔFMI in girls. Similarly, there were no significant associations between exposures (sedentary time and sleep duration) and ΔFFMI. However, in boys, there was a significant inverse association between sleep duration and ΔFMI. A difference of 1 h/night of baseline sleep was associated with 0.13 kg/m^2^ lower gain in FMI over follow-up in boys (Model 1), but evidence for this association diminished after adjustment for MVPA and depressive symptoms (Model 2). In all models, the potential for multicollinearity was low as variance inflation factors were ≤1.59 and tolerances ≥0.63. There was also no evidence of non-linear associations for sleep duration (*p*-value ≥0.17 in Models 1 and 2 for sleep^2^). All results were materially unchanged in sensitivity analyses.

## 4 Discussion

### 4.1 Sedentary time

This study found little evidence for a relationship between sedentary time and change in adolescent body fatness. Seven cross-sectional and three prospective studies have previously investigated objectively measured total sedentary time and its association with fatness in youth [6,31,32]. Consistently, cross-sectional studies have observed positive associations between variables in minimally adjusted models, but associations have been entirely attenuated when adjusted for engagement in MVPA. The results from longitudinal studies (two of which have specifically covered the adolescent age range of 12–18 years) are less consistent, with reported null [33] and positive associations [34]. It appears that, with few exceptions, total sedentary time is not independently associated with body fatness in youth, especially when accounting for the time spent in MVPA. Importantly, from a public health perspective, this should not be interpreted as meaning that sedentariness is irrelevant. Sedentary behaviours may under certain circumstances displace physical activity and sleep, and particular behaviours such as TV viewing do seem to exhibit positive associations with body fatness in youth, independent of the level of physical activity [6].

### 4.2 Sleep duration

To date, 10 longitudinal studies have investigated sleep length and body fatness in adolescence, and they report mixed findings [15]. Six studies report no association [35–40], whereas the remaining four studies have reported statistically significant inverse prospective associations between sleep duration and adiposity in both genders [41–44]. Most of these studies have been pre–post in design and have related change in BMI or obesity status to change in self-reported sleep [37,43] or sleep measured at the earlier time point [35,36,38–40,42]. Notably, however, Silva and colleagues [41] used a single night of polysomnography to objectively characterise sleep in 304 Americans aged 6–12 years, and used this information to predict the odds of obesity 5 years later. Adjusted for confounders including TV viewing, video use and baseline BMI, it was shown that compared to children sleeping >9 h/night, those sleeping <7.5 h/night had 3.3 (95% CI: 1.09–9.66) higher odds of obesity. The work by Mitchell and co-workers [44] also deserves mention as they incorporated a more refined study design. They followed adolescents every six months from 14 to 18 years of age, thereby providing a total of eight time points for sleep duration and BMI. Quantile regression showed that sleep duration was inversely associated with BMI at the 90th to the 25th BMI percentiles, with the magnitude of association diminishing toward the 25th percentile. We also found some evidence that sleep was inversely associated with ΔFMI in boys in a dose–response manner, but there was no indication that this association differed by the degree of baseline FMI (*p*-interaction = 0.31). Specifically, we observed that every 1-h difference in baseline nocturnal sleep was associated with 0.13 kg/m^2^ lower FMI gain in boys. The association was only borderline statistically significant, but when expressed relative to the mean ΔFMI over time (0.5 kg/m^2^ in boys) the association equates to 26% lower gain in body fatness per hour of baseline sleep, which is likely clinically relevant. Moreover, the magnitude of this association may be underestimated due to the short time frame over which we measured sleep (the same applies to the associations for sedentary time).

Like this investigation, other studies from diverse locations including America, Australia, Canada, Portugal, Turkey and Japan (all cross-sectional designs) have observed that sleep length is significantly inversely associated with adiposity (specifically BMI) in boys but not in girls [45–48], or that the magnitude of association is stronger in boys [49–51]. Two longitudinal studies, one situated in America [40] and the other in Portugal [39], have also reported inverse associations exclusively in boys, but only prior to adjusting for baseline BMI, the act of which made results non-significant. This has prompted speculation that girls may be more resilient to sleep debt [46] or that there may be gender differences in sleep architecture with adolescent girls experiencing proportionately more slow-wave sleep than boys, thereby reducing girls' sleep need [45]. Contradicting the second hypothesis, we found that girls on average slept longer than boys, which is a consistent finding in the literature.

Some of the longitudinal studies reporting inverse associations between sleep duration and change in adolescent BMI or obesity have described results that are independent of the self-reported physical activity level [42,44]. This could be attributed to residual confounding as a result of measurement error. In the current study, the association between sleep and ΔFMI in boys dissipated when adjusting for objectively measured MVPA (adjusting for sedentary time made no discernible change to the model). Adjusting for depression also attenuated the sleep–ΔFMI association, as has been reported by others [35]. Whether or not this means MVPA and depression mediate or confound the association between sleep and adolescent body fatness is a matter of interpretation. Adequate sleep may reduce obesity risk by preserving feelings of vitality and minimising fatigue, thereby augmenting physical activity levels (although we actually observed negative correlations between sleep duration and MVPA). Adequate sleep may also offset psychiatric co-morbidities, such as depression, which can lead to positive energy balance (in this study, 1 h/night of sleep was inversely cross-sectionally related to depressive symptoms in boys: *β* = −1.2, 95% CI −2.2 to −0.20, *p* = 0.023). Future investigations should formally test MVPA and depressive symptoms as potential mediating variables of the sleep–fatness association in adolescents, ideally with exposure, mediating, and outcome variables collected at multiple time points.

### 4.3 Strengths and weaknesses

This investigation included a relatively small sample that was leaner compared to ROOTS participants as a whole and also compared to the wider youth in Cambridgeshire, as identified from the Health Survey for England data [17]. Nonetheless, this would not have affected internal validity, and it is reassuring that our sample correctly reflected Cambridgeshire as a low ethnically diverse and prosperous county (82% of Cambridgeshire reside in middle- to high-SES locations) [16]. Due to homogeneity in our sample, attempts should be made to replicate our findings in larger and more diverse populations from different locations, preferably using repeated measures of sleep, sedentary time, body fatness, and potential mediators over follow-up. We advantageously used FMI as the outcome, not BMI, and measured sedentary time and sleep duration objectively. Although our method has not been validated against a criterion such as polysomnography performed in a free-living environment, actigraphy is an accepted means of measuring sleep length and we accompanied movement signals with heart rate by using a discrete monitor worn for multiple days. Heart rate declines significantly when transitioning from the waking state to sleep and the reverse occurs when waking and rising from bed [52]. Self-reported “usual” bed times were also used to inform our measurement of sleep, although we acknowledge that concurrent data from sleep diary would have been preferable. It is further unfortunate that our questionnaire did not enquire about sleep quality, which may influence obesity risk. We did, however, adjust for objectively measured MVPA and for depressive symptoms, which is a first. Future studies should consider doing the same, and they should also adjust for dietary components that may further confound or mediate associations.

To conclude, it seems that total sedentary time may not be independently associated with changes in adolescent body fat levels. Conversely, the association between sleep and change in adiposity may differ in boys and girls, with a potential inverse association in boys only. To clarify the nature of this association, studies are needed to demarcate MVPA and depression as either confounding or mediating factors.

## Conflict of interest

The authors declare that they have no conflict of interest and no financial relationships relevant to this article to disclose.

The ICMJE Uniform Disclosure Form for Potential Conflicts of Interest associated with this article can be viewed by clicking on the following link: http://dx.doi.org/10.1016/j.sleep.2015.02.532.

Conflict of interest ICMJE Form for Disclosure of Potential Conflicts of Interest form.

## Figures and Tables

**Table 1:** Demographic characteristics and baseline activity levels.^a^

	Boys (*n* = 213)	Girls (*n* = 291)	*p*-gender
Ethnicity (*n* (%) White)^b^	202 (95.3)^c^	266 (93.7)	0.44
Pubertal status (*n* (%) post-puberty)^d^	171 (80.3)	285 (97.9)	<0.001
Area-level SES (*n* (%))			
Low	35 (16.4)	37 (12.7)	
Middle	46 (21.6)	65 (22.3)	
High	132 (62.0)	189 (65.0)	0.50
PAEE (kJ/kg/day)	81.1 (30.7)^e^	63.4 (23.7)	<0.001
MVPA (min/day)	80.9 (61.7)	43.5 (40.6)	<0.001
Sedentary time (h/day)	5.8 ± 1.8^f^	6.4 ± 1.9	<0.001
Sleep duration (h/night)	8.0 ± 0.7	8.2 ± 0.8	<0.001
Depressive symptoms^d^	10 (10)	14 (14)	<0.001
Maternal BMI (kg/m^2^)^g^	24.6 (6.8)	24.4 (5.5)	0.92

a PAEE, physical activity energy expenditure; SES, socio-economic status; MVPA, moderate-to-vigorous physical activity.

b Data missing for eight participants (one boy and seven girls).

c Gender comparisons made by chi-squared tests (for all categorical variables).

d Measured at wave 0, approximately 6 months prior to baseline.

e Median (IQR) for all such variables with a skewed distribution and gender comparisons made by the Wilcoxon rank sum test.

f Mean ± SD, gender comparisons made by independent *t*-tests; there were no changes in results when clustering within schools was accounted for.

g Data available for 127 boys and 174 girls, only.

**Table 2:** Baseline and follow-up anthropometry and body composition.

	Boys (*n* = 213)		Girls (*n* = 291)		*p*-wave (whole sample)	*p*-wave*gender
	Baseline	Follow-up	Baseline	Follow-up		
Age (years)	15.0 ± 0.3^a^	17.5 ± 0.3	15.0 ± 0.3	17.5 ± 0.3	–	–
Weight (kg)	58.7 (13.2)^b^	67.4 (14.4)	53.4 (12.4)	57.1 (12.0)	<0.001	<0.001
Height (cm)	172.2 ± 7.5	178.7 ± 6.2	162.9 ± 5.8	164.7 ± 5.8	<0.001	<0.001
BMI (kg/m^2^)	19.8 (3.0)	21.3 (3.7)	20.1 (4.0)	21.2 (3.9)	<0.001	<0.001
Fat mass (kg)	8.4 (5.1)	10.3 (6.1)	13.2 (6.7)	15.1 (7.6)	<0.001	0.11
Fat mass index (kg/m^2^)	2.8 (1.7)	3.3 (1.9)	5.0 (2.5)	5.6 (2.5)	<0.001	0.61
Fat-free mass (kg)	50.3 (10.0)	58.1 (9.5)	40.2 (5.3)	42.5 (5.1)	<0.001	<0.001
Fat-free mass index (kg/m^2.5^)	12.9 (1.3)	13.5 (1.6)	11.9 (1.3)	12.2 (1.3)	<0.001	<0.001
Overweight and obese (*n* (%))^c^	33 (15.5)	42 (19.7)	46 (15.8)	54 (18.6)	0.16^d^	–

a Mean ± SD (all such values).

b Median (IQR) for all variables with skewed distribution.

c On the basis of International Obesity Task Force age- and gender-specific weight-for-height growth charts [30].

d Chi-squared test of baseline versus follow-up in boys and girls combined; all other comparisons were made using mixed-model ANOVA; there were no changes in results when the natural log of continuous variables with skewed distributions was used in analyses or when clustering within schools was accounted for.

**Table 3:** Prospective associations of sedentary time and sleep duration with changes in fat mass index (kg/m^2^) and fat-free mass index (kg/m^2.5^).^a^

	Boys (*n* = 213)		Girls (*n* = 291)	
	ΔFMI: *β* (95% CI)	*p*-value	ΔFMI: *β* (95% CI)	*p*-value
Model 1				
Sedentary time	0.039 (−0.037 to 0.11)	0.29	−0.060 (−0.16 to 0.043)	0.23
Sleep duration	−0.13 (−0.27 to −0.00054)	0.049	0.049 (−0.15 to 0.25)	0.61
Model 2				
Sedentary time	0.073 (−0.012 to 0.16)	0.087	−0.073 (−0.17 to 0.022)	0.12
Sleep duration	−0.11 (−0.26 to 0.043)	0.15	0.038 (−0.16 to 0.24)	0.69

	ΔFFMI: *β* (95% CI)		ΔFFMI: *β* (95% CI)	
Model 1				
Sedentary time	0.014 (−0.046 to 0.073)	0.64	−0.023 (−0.062 to 0.015)	0.22
Sleep duration	−0.088 (−0.18 to 0.0058)	0.064	0.0071 (−0.065 to 0.079)	0.84
Model 2				
Sedentary time	0.033 (−0.031 to 0.097)	0.29	−0.035 (−0.078 to 0.0082)	0.11
Sleep duration	−0.075 (−0.18 to 0.030)	0.15	−0.00014 (−0.072 to 0.071)	0.99

a FMI, fat mass index; FFMI, fat-free mass index; values represent the expected unit change in FMI (kg/m^2^) or FFMI (kg/m^2.5^) from baseline to follow-up per 1 h of baseline sedentary time or sleep duration (95% confidence intervals in parentheses); analyses performed using linear regression models with robust standard errors account for within-school clustering; Model 1 was adjusted for baseline age, area-level SES, pubertal status, season of activity measurement, weekday and weekend monitor wear time and follow-up duration; Model 2 was specified as per Model 1 but further adjusted for moderate-to-vigorous physical activity, depressive symptoms, sedentary time (when sleep duration was the independent variable of interest) or sleep duration (when sedentary time was the independent variable of interest); the results for sedentary time are restricted to 287 girls (four influential outliers were excluded from analyses).

## References

[1] Pate R.R., Mitchell J.A., Byun W. (2011). Sedentary behaviour in youth. Br J Sports Med.

[2] Nelson M.C., Neumark-Stzainer D., Hannan P.J. (2006). Longitudinal and secular trends in physical activity and sedentary behavior during adolescence. Pediatrics.

[3] Ng M., Fleming T., Robinson M. (2014). Global, regional, and national prevalence of overweight and obesity in children and adults during 1980–2013: a systematic analysis for the Global Burden of Disease Study 2013. Lancet.

[4] Biddle S.J., Gorely T., Marshall S.J. (2009). Is television viewing a suitable marker of sedentary behavior in young people?. Ann Behav Med.

[5] Pearson N., Biddle S.J.H. (2011). Sedentary behavior and dietary intake in children, adolescents, and adults a systematic review. Am J Prev Med.

[6] Ekelund U., Hildebrand M., Collings P.J. (2014). Physical activity, sedentary time and adiposity during the first two decades of life. Proc Nutr Soc.

[7] Beebe D.W. (2011). Cognitive, behavioral, and functional consequences of inadequate sleep in children and adolescents. Pediatr Clin North Am.

[8] Shochat T., Cohen-Zion M., Tzischinsky O. (2014). Functional consequences of inadequate sleep in adolescents: a systematic review. Sleep Med Rev.

[9] Matricciani L.A., Olds T.S., Blunden S. (2012). Never enough sleep: a brief history of sleep recommendations for children. Pediatrics.

[10] Matricciani L., Olds T., Petkov J. (2012). In search of lost sleep: secular trends in the sleep time of school-aged children and adolescents. Sleep Med Rev.

[11] Collishaw S., Maughan B., Natarajan L. (2010). Trends in adolescent emotional problems in England: a comparison of two national cohorts twenty years apart. J Child Psychol Psychiatry.

[12] Knutson K.L. (2012). Does inadequate sleep play a role in vulnerability to obesity?. Am J Hum Biol.

[13] Hart C.N., Cairns A., Jelalian E. (2011). Sleep and obesity in children and adolescents. Pediatr Clin North Am.

[14] Guidolin M., Gradisar M. (2012). Is shortened sleep duration a risk factor for overweight and obesity during adolescence? A review of the empirical literature. Sleep Med.

[15] Thind H., Davies S.L., Lewis T. (2014). Does short sleep lead to obesity among children and adolescents?: current understanding and implications. Am J Lifestyle Med.

[16] Goodyer I.M., Croudace T., Dunn V. (2010). Cohort profile: risk patterns and processes for psychopathology emerging during adolescence: the ROOTS project. Int J Epidemiol.

[17] Collings P.J., Wijndaele K., Corder K. (2014). Levels and patterns of objectively-measured physical activity volume and intensity distribution in UK adolescents: the ROOTS study. Int J Behav Nutr Phys Act.

[18] Wells J.C., Williams J.E., Chomtho S. (2010). Pediatric reference data for lean tissue properties: density and hydration from age 5 to 20 y. Am J Clin Nutr.

[19] Wells J.C.K., Williams J.E., Haroun D. (2009). Aggregate predictions improve accuracy when calculating metabolic variables used to guide treatment. Am J Clin Nutr.

[20] Wells J.C. (2001). A critique of the expression of paediatric body composition data. Arch Dis Child.

[21] Stegle O., Fallert S.V., MacKay D.J. (2008). Gaussian process robust regression for noisy heart rate data. IEEE Trans Biomed Eng.

[22] Brage S., Brage N., Franks P.W. (2004). Branched equation modeling of simultaneous accelerometry and heart rate monitoring improves estimate of directly measured physical activity energy expenditure. J Appl Physiol.

[23] Brage S., Westgate K., Wijndaele K. (2013). Evaluation of a method for minimising diurnal information bias in objective sensor data. International Conference on Ambulatory Monitoring of Physical Activity and Movement.

[24] Wolfson A.R., Carskadon M.A., Acebo C. (2003). Evidence for the validity of a sleep habits survey for adolescents. Sleep.

[25] CACI (2010). ACORN user guide. http://www.businessballs.com/freespecialresources/acorn-demographics-2010.pdf.

[26] Taylor S.J., Whincup P.H., Hindmarsh P.C. (2001). Performance of a new pubertal self-assessment questionnaire: a preliminary study. Paediatr Perinat Epidemiol.

[27] Daviss W.B., Birmaher B., Melhem N.A. (2006). Criterion validity of the mood and feelings questionnaire for depressive episodes in clinic and non-clinic subjects. J Child Psychol Psychiatry.

[28] Goodyer I.M., Bacon A., Ban M. (2009). Serotonin transporter genotype, morning cortisol and subsequent depression in adolescents. Br J Psychiatry.

[29] Patel S.R., Hu F.B. (2008). Short sleep duration and weight gain: a systematic review. Obesity (Silver Spring).

[30] Cole T.J., Bellizzi M.C., Flegal K.M. (2000). Establishing a standard definition for child overweight and obesity worldwide: international survey. BMJ.

[31] Hjorth M.F., Chaput J.P., Ritz C. (2014). Fatness predicts decreased physical activity and increased sedentary time, but not vice versa: support from a longitudinal study in 8- to 11-year-old children. Int J Obes.

[32] Chaput J.P., Leduc G., Boyer C. (2014). Objectively measured physical activity, sedentary time and sleep duration: independent and combined associations with adiposity in Canadian children. Nutr Diabetes.

[33] Kwon S., Burns T.L., Levy S.M. (2013). Which contributes more to childhood adiposity-high levels of sedentarism or low levels of moderate-through-vigorous physical activity? The Iowa Bone Development Study. J Pediatr.

[34] Mitchell J.A., Pate R.R., Beets M.W. (2013). Time spent in sedentary behavior and changes in childhood BMI: a longitudinal study from ages 9 to 15 years. Int J Obes.

[35] Calamaro C.J., Park S., Mason T.B.A. (2010). Shortened sleep duration does not predict obesity in adolescents. J Sleep Res.

[36] Bell J.F., Zimmerman F.J. (2010). Shortened nighttime sleep duration in early life and subsequent childhood obesity. Arch Pediatr Adolesc Med.

[37] Lytle L.A., Murray D.M., Laska M.N. (2013). Examining the longitudinal relationship between change in sleep and obesity risk in adolescents. Health Educ Behav.

[38] Snell E.K., Adam E.K., Duncan G.J. (2007). Sleep and the body mass index and overweight status of children and adolescents. Child Dev.

[39] Araujo J., Severo M., Ramos E. (2012). Sleep duration and adiposity during adolescence. Pediatrics.

[40] Storfer-Isser A., Patel S.R., Babineau D.C. (2012). Relation between sleep duration and BMI varies by age and sex in youth age 8–19. Pediatr Obes.

[41] Silva G.E., Goodwin J.L., Parthasarathy S. (2011). Longitudinal association between short sleep, body weight, and emotional and learning problems in Hispanic and Caucasian children. Sleep.

[42] Seegers V., Petit D., Falissard B. (2011). Short sleep duration and body mass index: a prospective longitudinal study in preadolescence. Am J Epidemiol.

[43] Rutters F., Gerver W.J., Nieuwenhuizen A.G. (2010). Sleep duration and body-weight development during puberty in a Dutch children cohort. Int J Obes.

[44] Mitchell J.A., Rodriguez D., Schmitz K.H. (2013). Sleep duration and adolescent obesity. Pediatrics.

[45] Knutson K.L. (2005). Sex differences in the association between sleep and body mass index in adolescents. J Pediatr.

[46] Eisenmann J.C., Ekkekakis P., Holmes M. (2006). Sleep duration and overweight among Australian children and adolescents. Acta Paediatr.

[47] Ramos E., Barros H. (2007). Family and school determinants of overweight in 13-year-old Portuguese adolescents. Acta Paediatr.

[48] Ozturk A., Mazicioglu M.M., Poyrazoglu S. (2009). The relationship between sleep duration and obesity in Turkish children and adolescents. Acta Paediatr.

[49] Sekine M., Yamagami T., Handa K. (2002). A dose-response relationship between short sleeping hours and childhood obesity: results of the Toyama Birth Cohort Study. Child Care Health Dev.

[50] Chaput J.P., Brunet M., Tremblay A. (2006). Relationship between short sleeping hours and childhood overweight/obesity: results from the ‘Quebec en Forme’ Project. Int J Obes.

[51] Shi Z.M., Taylor A.W., Gill T.K. (2010). Short sleep duration and obesity among Australian children. BMC Public Health.

[52] Somers V.K., Dyken M.E., Mark A.L. (1993). Sympathetic-nerve activity during sleep in normal subjects. N Engl J Med.

